# Closing the Satisfaction Gap: Medial UKA and Cruciate-Retaining TKA Yield Comparable 1-Year Patient-Reported Outcome Measures

**DOI:** 10.2106/JBJS.OA.25.00345

**Published:** 2026-02-17

**Authors:** Shlok V. Patel, Khaled A. Elmenawi, Shujaa T. Khan, Lakshmi Spandana Gudapati, Chao Zhang, Nicolas S. Piuzzi

**Affiliations:** 1 Department of Orthopaedic Surgery, Cleveland Clinic, Cleveland, Ohio

## Abstract

**Background:**

Medial unicompartmental knee arthroplasty (mUKA) has historically yielded higher patient satisfaction than total knee arthroplasty (TKA) for isolated medial compartment osteoarthritis, but recent advances in robotic mUKA and contemporary TKA alignment strategies have narrowed this gap in outcomes. Pragmatic comparisons of mUKA and modern cruciate-retaining (CR) TKA focusing on 1-year patient-reported outcome measures (PROMs) and healthcare utilization are limited.

**Methods:**

We analyzed 2771 CR-TKA and 696 mUKA patients between 2016 and 2023 using a prospective registry (median age: 68 years; 55% women; 89% White). Baseline and 1-year PROMs including Knee Injury and Osteoarthritis Outcome Score (KOOS) for Pain, Physical Function Shortform (PS), Joint Replacement (JR), and Veterans RAND 12-Item Mental Component Summary (VR-12 MCS) were collected. Clinically meaningful improvement was assessed using minimal clinically important difference (MCID) and Patient Acceptable Symptom State (PASS) thresholds. Healthcare utilization included length of stay (LOS), discharge disposition, 90-day readmissions, 1-year reoperations, and 1-year mortality. Multivariable regression models compared outcomes between mUKA and CR-TKA.

**Results:**

At 1 year, 82% to 93% of mUKA and 84%–94% of CR-TKA patients achieved MCID thresholds for KOOS subscales. PASS thresholds achievement ranged from 69% to 71% for mUKA and 68% to 73% for CR-TKA. Satisfaction was reported by 83% of mUKA and 87% of CR-TKA patients. In multivariable models, CR-TKA compared with mUKA showed no significant associations with 1-year KOOS-Pain, PS, JR, failure to achieve MCID and PASS thresholds, and risk of dissatisfaction. CR-TKA was significantly associated with LOS ≥3 days and nonhome discharge.

**Conclusions:**

At 1 year, CR-TKA and mUKA demonstrated comparable PROMs and satisfaction. CR-TKA was associated with a longer LOS and nonhome discharge. Longer-term comparative data on revision, survivorship, and cost are needed to refine patient selection and indications.

**Level of Evidence:**

Level III. See Instructions for Authors for a complete description of levels of evidence.

## Introduction

Medial unicompartmental knee arthroplasty (mUKA) has historically demonstrated higher patient satisfaction compared with total knee arthroplasty (TKA), particularly in patients with isolated medial compartment osteoarthritis, which accounts for an estimated 25% to 47% of arthroplasty candidates^1-3^. Traditionally, mUKA was offered selectively, guided by strict selection criteria^3^. However, recent advancements in robotic-assisted UKA have reduced early revision rates and enhanced reproducibility compared with manual approaches, leading to broader adoption among surgeons^4-7^.

In parallel, TKA has evolved with advancements in alignment strategies^8-12^. Notably, kinematically aligned knees have demonstrated comparable or superior patient-reported outcome measures (PROMs) compared with mechanical alignment^9-12^. With these developments in both mUKA and TKA, evidence now suggests that the gap in PROMs between procedures may be smaller than once assumed. The randomized TOPKAT trial reported broadly similar 5-year PROMs for appropriately selected patients undergoing UKA or TKA^13^.

While both mUKA and TKA are established surgical treatments, procedural choice remains largely influenced by surgical indications, patient characteristics and, surgeon preference^3^. Medial-UKA may confer short-term benefits over TKA, namely, quicker recovery, shorter hospital stay, and lower postoperative morbidity and perioperative costs^14-16^. However, these early advantages of mUKA must be weighed against the long-term durability: a systematic review reported 15-year survivorship of 89% for TKA versus 70% for UKA^17^. These durability differences extend to implant design type, with mobile-bearing UKA exceeding fixed-modular UKA in 15-year cumulative revision rate (23 vs. 16%)^18,19^.

Pragmatic, real-world direct comparisons between mUKA and cruciate-retaining (CR) TKA remain limited. We compared mUKA and CR-TKA procedures evaluating 1-year PROMs and healthcare utilization outcomes. Because follow-up duration is 1 year, this study does not address survivorship and longer-term healthcare utilization.

## Methods

### Study Design

This study analyzed a prospective patient cohort identified from the institutional database of a large tertiary academic center. Orthopaedic Minimal Data Set Episode of Care cohort^8,20-24^, a previously validated data collection platform, records PROMs in a consecutive series of patients using Research Electronic Data Capture (REDCap) software. Patients undergoing primary elective TKA and UKA between January 2016 and December 2023 were considered for inclusion in the study. All procedures were performed at a high-volume tertiary academic center by fellowship-trained adult reconstruction surgeons. Procedural selection was based on standard contemporary indications. In general, mUKA was offered to patients with predominantly isolated, correctable medial compartment osteoarthritis and preserved cruciate and collateral ligaments, whereas CR-TKA was chosen for those with bicompartmental or tricompartmental disease, ligamentous insufficiency, severe fixed deformity, or inflammatory arthritis.

### Patient Population

**Fig. 1:**
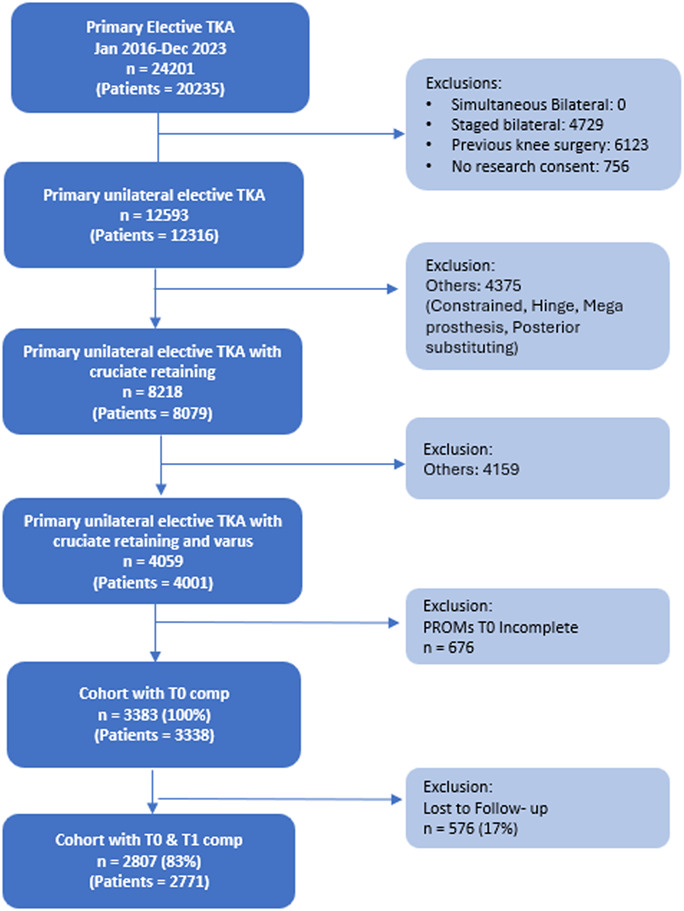
STROBE diagram for CR-TKA patient cohort. CR-TKA = cruciate-retaining-total knee arthroplasty, and STROBE = strengthening the reporting of observational studies in epidemiology.

**Fig. 2:**
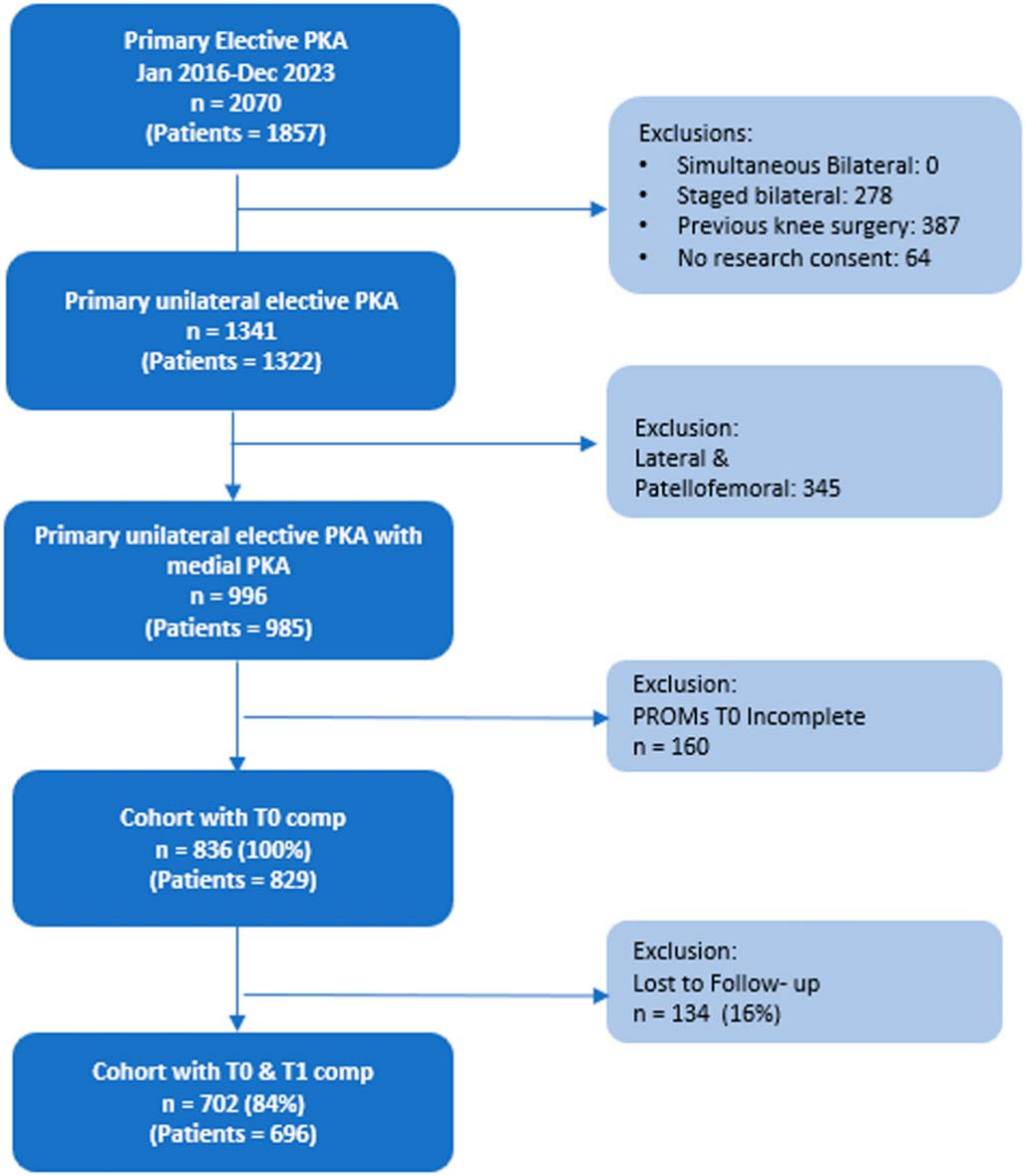
STROBE diagram for mUKA patient cohort. CR-TKA = cruciate-retaining-total knee arthroplasty, and mUKA = Medial unicompartmental knee arthroplasty.

We identified 24,201 primary TKAs and 2070 primary UKAs between 2016 and 2023. Patients who underwent simultaneous or staged bilateral procedures, constrained, hinge, megaprosthesis or posterior-substituting TKAs, lateral or patellofemoral UKAs, prior knee surgery, as well as those with incomplete datasets, were excluded. Only patients with complete preoperative and postoperative PROMs were included. Approximately 17% of patients in the TKA group and 16% in the UKA group were lost to follow-up. We restricted inclusion to varus-aligned CR-TKA to minimize heterogeneity from lateral or patellofemoral-predominant disease and differing implant indications. This left 2771 CR-TKA and 696 mUKA patients for analysis (Figs. 1 and 2). Overall, the median age of the entire cohort was 68 years (62-73); 55% of patients were women, and 89.3% were White (Table I).

**TABLE I:** Patient Characteristics of mUKA and CR-TKA Cohorts

Variable	Level	All (n = 3,467)	mUKA (n = 696)	CR-TKA (n = 2,771)	p
Age		68.0 (62.0; 73.0)	67.0 (60.0; 73.0)	68.0 (62.0; 73.0)	0.082
Sex	Female	1908 (55.0%)	369 (53.0%)	1,539 (55.5%)	0.249
	Male	1,559 (45.0%)	327 (47.0%)	1,232 (44.5%)	
BMI		30.5 (27.1; 35.0)	29.0 (25.8; 32.7)	31.0 (27.4; 35.5)	**<0.001**
Education		14.0 (12.0; 16.0)	14.0 (12.0; 16.0)	14.0 (12.0; 16.0)	0.825
ADI		42.0 (25.0; 61.0)	42.0 (23.0; 60.0)	42.0 (26.0; 61.0)	0.126
Race	White	3,025 (89.3%)	614 (90.4%)	2,411 (89.1%)	0.338
	Non-White	361 (10.7%)	65 (9.57%)	296 (10.9%)	
Smoking	Never	2034 (58.7%)	385 (55.3%)	1,649 (59.5%)	0.051
	Quit >6 mo	1,207 (34.8%)	260 (37.4%)	947 (34.2%)	
	Quit 0-6 mo	82 (2.37%)	13 (1.87%)	69 (2.49%)	
	Current	144 (4.15%)	38 (5.46%)	106 (3.83%)	
CCI	0	1746 (50.8%)	396 (57.6%)	1,350 (49.1%)	**0.001**
	1	692 (20.1%)	122 (17.7%)	570 (20.7%)	
	2	478 (13.9%)	90 (13.1%)	388 (14.1%)	
	3+	520 (15.1%)	80 (11.6%)	440 (16.0%)	
Insurance	Other	1,232 (38.2%)	262 (40.9%)	970 (37.5%)	0.126
	Medicaid/Medicare	1,996 (61.8%)	379 (59.1%)	1,617 (62.5%)	
Implant manufacturer	Biomet	274 (7.90%)	270 (38.8%)	4 (0.14%)	**<0.001**
	DePuy	1 (0.03%)	0 (0.00%)	1 (0.04%)	
	Smith & Nephew	63 (1.82%)	22 (3.16%)	41 (1.48%)	
	Stryker	2,343 (67.6%)	145 (20.8%)	2,198 (79.3%)	
	Zimmer	779 (22.5%)	252 (36.2%)	527 (19.0%)	
	Other	7 (0.20%)	7 (1.01%)	0 (0.00%)	
Robotic-assisted procedures		1,039 (35%)	107 (18%)	932 (40%)	-
Baseline KOOS-pain		44.4 (33.3; 52.8)	44.4 (33.3; 55.6)	41.7 (33.3; 52.8)	**0.031**
Baseline KOOS-PS		53.9 (45.6; 59.7)	53.9 (45.6; 61.4)	53.9 (45.6; 59.7)	**0.018**
Baseline KOOS-JR		47.5 (39.6; 54.8)	47.5 (42.3; 57.1)	47.5 (39.6; 54.8)	**0.001**
Phenotype	P− PS− MCS−	830 (24.0%)	158 (22.7%)	672 (24.3%)	0.335
	P+ PS+ MCS+	868 (25.1%)	194 (27.9%)	674 (24.3%)	
	P+ PS+ MCS−	524 (15.1)	113 (16.2)	411 (14.8)	
	P+ PS− MCS+	176 (5.08%)	35 (5.03%)	141 (5.09%)	
	P+ PS− MCS−	167 (4.82%)	34 (4.89%)	133 (4.80%)	
	P− PS+ MCS+	197 (5.69%)	35 (5.03%)	162 (5.85%)	
	P− PS+ MCS−	211 (6.09%)	44 (6.32%)	167 (6.03%)	
	P− PS− MCS+	492 (14.2%)	83 (11.9%)	409 (14.8%)	

ADI = area deprivation index, BMI = body mass index, CCI = Charlson Comorbidity Index, CR-TKA = cruciate-retaining-total knee arthroplasty, JR = joint replacement, KOOS, Knee injury and Osteoarthritis Outcome Score, MCS = Mental Composite Score, mUKA = medial unicompartmental knee arthroplasty, P = Pain, PS = physical function shortform, and VR-12 = veteran RAND-12,

Continuous variables presented as median (IQR). Categorical variables presented as N (column %). Statistical significance was defined as* P *value < 0.05.

Among TKAs, 79% received a Stryker Triathlon CR and 19% a Zimmer Persona CR, with the remaining ≈2% using other systems. Medial UKAs were performed mainly with Biomet Oxford (≈35%), Stryker Mako-based or PKR-based partial knees (≈21%), and Zimmer ZUK unicompartmental knees (≈20%), with ≈23% using other designs.

### Outcomes

The primary objective was to compare 1-year PROMs and attainment of minimum clinically important difference (MCID) and patient acceptable symptom state (PASS) thresholds between mUKA and CR-TKA. PROMs included Knee injury and Osteoarthritis Outcome Score (KOOS) Pain, KOOS Physical Function Shortform (KOOS-PS), KOOS Joint Replacement (KOOS-JR), and Veterans RAND 12-Item Health Survey Mental Component Score (VR-12 MCS)^25-31^. Clinically meaningful improvement was defined as achieving established MCID or PASS thresholds for KOOS subdomains^32^. Failure to reach the threshold was modeled as the outcome of interest. MCID cutoffs were 7.99 for KOOS-Pain, 8.04 for KOOS-PS, and 6.76 for KOOS-JR; PASS cutoffs were 77.7 for KOOS-Pain and 70.3 for KOOS-PS and KOOS-JR^33,34^. Patient satisfaction was collected at 1 year using the question: Taking into account all the activity you have during your daily life, your level of pain, and also your activity limitations and participation restrictions, do you consider the current state of your knee satisfactory? with patients answering “yes” or “no”^35^. This question was adapted from previous literature^36-38^.

Our secondary objective involved comparing healthcare utilization between cohorts using discharge disposition, length of stay (LOS), 90-day readmission, reoperation, and mortality as metrics. Reoperation was defined as any procedure performed on the same knee and same laterality between postoperative days 1 and 365 after TKA, excluding manipulation and closed reduction.

### Statistical Analysis

Continuous variables were summarized as medians with interquartile ranges and categorical variables as counts and percentages. The Wilcoxon rank-sum test was applied for continuous variables and the χ^2^ test for categorical variables. Multivariable logistic models adjusted for age, sex, body mass index, education, race, smoking status, Area Deprivation Index, insurance type, Charlson Comorbidity Index, and baseline PROM phenotypes were used to compare PROMs, clinically meaningful improvements, and healthcare utilization between cohorts. PROM phenotypes were defined using preoperative KOOS-Pain, KOOS-PS, and VR-12 MCS scores, classifying each domain as above (+) or below (–) the cohort median to yield 8 categories^39-41^. Associations were reported as odds ratios (OR) and regression coefficients (β). Analyses were performed using R (version 4.0) with statistical significance set at 2-sided p < 0.05.

## Ethics Statement

IRB approval was obtained from the Cleveland Clinic Institutional Review Board (IRB 06-196). Data were obtained from a prospectively maintained institutional registry: Orthopaedic Minimal Data Set Episode of Care registry.

## Results

### Baseline and 1-Year PROMs

While baseline KOOS-Pain, PS, and JR scores were slightly better in the mUKA cohort than in the CR-TKA group, no significant differences in 1-year KOOS-Pain, PS, and JR scores were noted between cohorts (P > 0.05) (Tables I and II).

**TABLE II:** Outcomes of mUKA and CR-TKA Cohorts

Outcomes	Level	All (n = 3,467)	mUKA (n = 696)	CR-TKA (n = 2,771)	p
1-year PROM scores					
KOOS-pain		88.9 (75.0; 97.2)	87.5 (75.0; 97.2)	88.9 (75.0; 97.2)	0.423
KOOS-PS		75.1 (66.4; 85.2)	78.0 (66.4; 85.2)	75.1 (66.4; 85.2)	0.386
KOOS-JR		76.3 (68.3; 92.0)	76.3 (66.0; 92.0)	76.3 (68.3; 92.0)	0.564
Baseline-1 yr PROM score change					
KOOS-pain difference		41.7 (27.8; 52.8)	38.9 (25.0; 52.8)	41.7 (27.8; 52.8)	**0.008**
KOOS-PS difference		23.6 (12.8; 35.6)	22.2 (12.4; 34.3)	23.7 (13.2; 35.6)	0.120
KOOS-JR difference		29.8 (18.5; 42.0)	28.4 (16.3; 39.7)	29.9 (18.6; 42.0)	**0.002**
Clinically meaningful thresholds (%)					
PASS		2,921 (85.9%)	572 (83.4%)	2,349 (86.6%)	**0.036**
KOOS-pain MCID	Failure	222 (6.44%)	50 (7.27%)	172 (6.23%)	0.365
	Improved	3,227 (93.6%)	638 (92.7%)	2,589 (93.8%)	
KOOS-PS MCID	Failure	552 (16.5%)	123 (18.2%)	429 (16.1%)	0.19
	Improved	2,793 (83.5%)	551 (81.8%)	2,242 (83.9%)	
KOOS-JR MCID	Failure	264 (8.64%)	61 (9.98%)	203 (8.31%)	0.215
	Improved	2,791 (91.4%)	550 (90.0%)	2,241 (91.7%)	
PASS threshold for KOOS-Pain	Failure	937 (27.2%)	198 (28.8%)	739 (26.8%)	0.31
	Improved	2,512 (72.8%)	490 (71.2%)	2022 (73.2%)	
PASS threshold for KOOS-PS	Failure	1,059 (31.6%)	204 (30.3%)	855 (32.0%)	0.417
	Improved	2,288 (68.4%)	470 (69.7%)	1818 (68.0%)	
PASS threshold for KOOS-JR	Failure	897 (29.4%)	188 (30.8%)	709 (29.0%)	0.418
	Improved	2,159 (70.6%)	423 (69.2%)	1736 (71.0%)	
Healthcare utilization					
LOS ≥3		273 (7.88%)	5 (0.72%)	268 (9.68%)	**<0.001**
DD	Home/home health care	3,298 (95.2%)	691 (99.3%)	2,607 (94.2%)	**<0.001**
	Nonhome	166 (4.79%)	5 (0.72%)	161 (5.82%)	
90-d readmission		197 (5.69%)	23 (3.30%)	174 (6.28%)	**0.003**
90-d reoperation		95 (3.43%)	0 (0%)	95 (3.43%)	-
Mortality		0 (0%)	0 (0%)	0 (0%)	-

CR-TKA = cruciate-retaining-total knee arthroplasty, DD = discharge disposition, JR = joint replacement, KOOS = Knee injury and Osteoarthritis Outcome Score, LOS = length of stay, MCID = minimal clinically important difference, mUKA = medial unicompartmental knee arthroplasty, PASS = patient acceptable symptom state, and PS = physical function Shortform.

Continuous variables presented as median (IQR). Categorical variables presented as N (column %). Statistical significance was defined as* P *value < 0.05.

In the multivariable adjusted model, CR-TKA showed no significant associations compared with mUKA with 1-year KOOS scores: KOOS-Pain (β = 0.26), KOOS-PS (β = −0.41), and KOOS-JR (β = 0.48; all p > 0.55) (Table III).

**TABLE III:** Multivariate Analysis of CR-TKA Versus mUKA PROMS and Healthcare Utilization

Outcomes	β or (CR-TKA vs. mUKA)	95% CI	p
1-y PROM scores			
KOOS-pain	0.26	−1.42 to 1.94	0.763
KOOS-PS	−0.41	−1.92 to 1.10	0.592
KOOS-JR	0.48	−1.09 to 2.05	0.551
Clinically meaningful improvement thresholds (failure)			
MCID threshold for KOOS-pain	1.07	0.71 to 1.64	0.739
MCID threshold for KOOS-PS	0.9	0.68 to 1.20	0.483
MCID threshold for KOOS-JR	0.96	0.66 to 1.38	0.816
PASS threshold for KOOS-pain	0.9	0.71 to 1.14	0.392
PASS threshold for KOOS-PS	1.09	0.86 to 1.38	0.491
PASS threshold for KOOS-JR	0.93	0.73 to 1.18	0.543
PASS (satisfaction)	0.8	0.60 to 1.07	0.133
Healthcare utilization			
LOS ≥3 d	12.93	4.09 to 40.84	**<0.001**
DD (nonhome)	4.39	1.76 to 10.95	**0.002**
90-d readmission	1.71	0.99 to 2.94	0.055

β = regression coefficient (continuous outcomes), CI = confidence interval, DD = discharge disposition, JR = joint replacement, LOS = length of Stay, MCID = minimal clinically important difference; OR = odds ratio (binary outcomes), PS = physical function Shortform, and PASS = patient acceptable symptom state. Statistical significance was defined as* P *value < 0.05.

### Clinically Meaningful Improvement

At 1 year, the proportion of patients achieving MCID thresholds was similar between CR-TKA and mUKA groups for KOOS-Pain (93.8% vs. 92.7%), KOOS-PS (83.9% vs. 81.8%), and KOOS-JR (91.7% vs. 90%, all P values >0.19). PASS threshold achievement rates were also comparable between CR-TKA and mUKA for KOOS-Pain (73.2% vs. 71.2%), KOOS-PS (68% vs. 69.7%), and KOOS-JR (71% vs. 69.2%, all p values >0.31). The proportion of patients reporting satisfaction was significantly higher following CR-TKA than mUKA (86.6% vs. 83.4%, p value = 0.036).

After adjusting for confounders, CR-TKA was not significantly correlated with the odds of failure to achieve MCID thresholds for KOOS-Pain (OR = 1.07), KOOS-PS (OR = 0.90), and KOOS-JR (OR = 0.96; all p > 0.48) compared with mUKA. Similarly, failure to achieve PASS thresholds was not significantly associated with CR-TKA compared with mUKA for KOOS-Pain (OR = 0.90), KOOS-PS (OR = 1.09), or KOOS-JR (OR = 0.93; all p > 0.39). In addition, the likelihood of dissatisfaction was not significantly associated with CR-TKA compared with mUKA (OR = 0.80; p = 0.133) (Table III).

### Healthcare Utilization

After adjusting for confounders, CR-TKA was associated with increased odds of LOS ≥3 days (OR: 12.93; p < 0.001) and nonhome discharge (OR: 4.39; p = 0.002) compared with mUKA. The CR-TKA cohort was not significantly associated with 90-day readmission (OR: 1.71; p = 0.055) relative to mUKA (Table III).

## Discussion

At 1 year, KOOS (Pain, PS, and JR), achievement of MCID and PASS thresholds for KOOS subdomains, and satisfaction did not show a significant difference between CR-TKA and mUKA. In addition, CR-TKA was associated with higher odds of longer LOS and nonhome discharge. Accordingly, surgeons can expect comparable 1-year PROMs and clinically meaningful improvements with either procedure. As such, these findings support a narrowing gap in PROMs and satisfaction between the procedures and inform procedural selection and shared decision-making based on patient characteristics, goals, and presenting pathology. Longer-term studies focused on survivorship, cost, and revision risk are required.

Laoruengthana et al., in a propensity score–matched comparison of mUKA and CR-TKA, demonstrated that although early postoperative pain scores and opioid requirements were similar between groups, patients undergoing mUKA achieved greater knee flexion, performed straight-leg raises more frequently and to higher degrees, and ambulated independently sooner, indicating superior immediate functional recovery^19^. However, our study suggests that these early benefits may not translate to superior 1-year PROMs. Most previous studies have focused predominantly on posterior-stabilized or unspecified TKA designs, thereby limiting generalizability to CR-TKAs. A large meta-analysis of 29 studies recorded significantly better PROMs after mUKA than after TKA, with a standard mean difference of –0.19 from 6 randomized controlled trials and −0.19 from 18 cohort studies^2^. While many previous studies report superior PROMs with mUKA versus TKA, the TOPKAT randomized trial found no difference in 5-year Oxford Knee Scores between groups, aligning with our results^13^.

Patients undergoing primary TKA are expected to have higher healthcare utilization than mUKA patients. This likely stems from the fact that TKAs are more invasive, with longer incisions, greater blood loss, and extensive soft-tissue dissection compared with mUKAs^42^. Laoruengthana et al. found that mUKA was associated with a shorter LOS and 12.43% lower hospitalization costs compared with CR-TKA^19^. These findings corroborate our results, highlighting increased healthcare utilization among CR-TKA patients compared with mUKA. This is consistent with prior reports indicating quicker recovery following mUKA than TKA^43^. Nonetheless, there is a need for longer-term studies to directly compare healthcare utilization and cost-effectiveness between both procedures.

Patients selected for either CR-TKA or mUKA can expect similar 1-year patient-reported pain relief and function. Our results counter the prevailing belief that mUKA offers superior PROMs and satisfaction and highlight an emerging overlap in indications for mUKA and CR-TKA. Rather than a one-size-fits-all approach, our findings emphasize the importance of a tailored surgical approach to procedural selection. Expected lifetime revision risk and willingness to undergo possible reoperation should be weighed in selection, given higher long-term revision rates after UKA^17^. Technical considerations should guide procedure choice: mUKA most commonly fails from progression to other compartments, polyethylene wear, or tibial component loosening and revision typically entails conversion to a primary-type TKA with contained bone loss; by contrast, CR-TKA more often fails due to instability, stiffness, or patellofemoral complications and usually requires revision-grade implants with greater resource demands^3^. Comparative analyses should therefore account for these differential revision pathways and their associated morbidity, operative demands, and costs when comparing mUKA and CR-TKA^4,14^.

Surgeon and center volume may influence mUKA outcomes, institutional expertise should therefore factor into procedural selection^44^. CR-TKA may be more suitable for patients who do not fit the selection criteria for mUKA (ACL/MCL insufficiency, severe fixed deformity, tricompartmental disease, and inflammatory arthritis). However, patients who require a quicker return to daily function, such as younger individuals, athletes, working professionals, or those with caregiving responsibilities, may be better suited for a mUKA, particularly younger, athletic patients with high functional demands in whom a higher functional ceiling and more rapid return to sports have been reported^45^. The choice between CR-TKA and mUKA should be guided by a shared decision-making framework that accounts for patient-specific factors^15^. Our findings inform resource planning by highlighting differences in healthcare utilization during 1-year follow-up between the 2 procedures. However, any initial cost advantage from shorter hospital stays and lower index costs with UKA may narrow once long-term revision rates are considered.

This study has some limitations. The analysis did not adjust for radiographic disease severity that may have influenced selection and introduced residual confounding. The single-institution setting may limit external validity. The study period spanned major changes in guidelines, care pathways, technology, and reimbursement that likely influenced selection, perioperative care, discharge disposition, and outcomes, but we could not fully adjust for these temporal shifts. Thus, differences in LOS, discharge patterns, and 90-day utilization may reflect evolving practice rather than intrinsic contrasts between procedures. Unmeasured confounders such as discharge and rehabilitation protocols may have influenced outcomes. We could not account for surgeon-specific factors that may confound selection and outcomes. Furthermore, selection bias likely exists as the selection criteria for mUKA vs CR-TKA vary widely. Finally, the 1-year follow-up may not capture long-term differences in reoperation or late complications, warranting longitudinal studies. Although prior evidence indicates that PROMs tend to plateau after 1 year, additional research is warranted to confirm the long-term durability of these associations^46,47^. Moreover, our cohort predominantly comprised older patients, so these findings may not generalize to younger, high-demand or athletic individuals, in whom mUKA may confer a higher functional ceiling and superior activity or return-to-sport outcomes compared with TKA^45^.

## Conclusion

This study found no significant differences in 1-year PROMs or rates of achieving clinically meaningful improvement thresholds between mUKA and CR-TKA. mUKA was associated with shorter hospital stays and more frequent home discharges. These results align with existing evidence that the PROMs and satisfaction gap between the procedures is narrowing. Our findings reflect short-term results in the treated cohorts and do not establish conclusions about long-term comparability. Prospective, randomized studies in larger, more diverse populations are needed to evaluate long-term outcomes, implant survivorship, and healthcare utilization between both procedures.

## Funding

This study received no external funding.

## APPENDIX. Cleveland Clinic Adult Reconstruction Research (CCARR) Group

Cleveland Clinic Adult Reconstruction Research (CCARR) Group consists of: Trevor G. Murray, MD, Robert M. Molloy, MD, Viktor E. Krebs, MD, Nicholas R. Scarcella, MD, Michael Erossy, MD, Alexander L. Roth, MD, Michael R. Bloomfield, MD, John P. McLaughlin, DO, Peter A. Surace, MD, Matthew Deren, MD.
